# Cyanobacterial diversity of biological soil crusts and soil properties in karst desertification area

**DOI:** 10.3389/fmicb.2023.1113707

**Published:** 2023-03-13

**Authors:** Qian Chen, Ni Yan, Kangning Xiong, Jiawei Zhao

**Affiliations:** ^1^School of Karst Science, Guizhou Normal University, Guiyang, China; ^2^State Engineering Technology Institute for Karst Desertification Control, Guizhou Normal University, Guiyang, China; ^3^School of Life Science, Guizhou Normal University, Guiyang, China

**Keywords:** biological soil crusts, cyanobacteria, species diversity, soil properties, karst desertification

## Abstract

As important components of the biological soil crusts (BSCs) and of the primary stage of crust succession, cyanobacterial communities occupy an important ecological niche and play an important ecological role in desertification areas. In this study, we focused on the karst desertification area, which also belongs to the same category of desertification, and selected three study areas, Guanling-Zhenfeng Huajiang (HJ), Bijie Salaxi (SLX), and Shibing (SB), in the Guizhou Plateau, which represents the overall ecological environment of South China karst, to conduct surveys on the diversity of BSC species and soil properties. Analysis of the cyanobacterial communities and physicochemical properties using the Shannon-Wiener diversity index, principal component analysis, and redundancy analysis revealed that: (1) The three study areas had common cyanobacterial species, with a total of 200 species distributed across 22 genera, 2 classes, 5 orders, and 6 families belonging to the Oscillatoriales (39%), Scytonematales (24.5%), Chroococcales (23%), Nostocales (11.5%), and Rivulariales (2%), (2) The number of species increased with the intensity of karst desertification—while Oscillatoriaceae was the dominant family in HJ and moderate–severe desertification areas, Chroococcaceae and Scytonemataceae were dominant in the mild and potential desertification areas SLX and SB, (3) The Shannon-Wiener diversity indices followed the trend: SLX (3.56) > SB (3.08) > HJ (3.01), indicating that the species were more evenly distributed in mild desertification, (4) In the carbonate background, shrubland harbored the largest number of cyanobacterial species compared to grassland, bare land, and arbor woodland; however, the highest number was documented in arbor woodland in dolomite karst, (5) The soil is weathered limestone or yellow soil in all three areas, with pH ranging from 5.73 to 6.85, fine sand dominated, and soil nutrients increased with the intensity of desertification, and (6) Redundancy analysis showed that organic carbon, soil moisture content (0–5 cm), and total nitrogen substantially influenced cyanobacterial diversity. These results reveal that differences in soil nutrient content play an important role in regulating the cyanobacterial diversity and composition, thereby establishing a foundation for further research and application of soil ecological restoration of cyanobacteria in BSCs of karst desertification areas.

## 1. Introduction

BSCs are an important component of desert ecosystems that have received widespread attention in desertification areas, it refers to the plant–soil complexes formed by different proportions of cyanobacteria, algae, lichens, mosses, and microorganisms cementing soil particles (Belnap and Lange, 2003; Belnap and Miller, 2004). It covers 12% of the global land area, with some arid regions covering more than 70% (Belnap and Lange, 2001). Cyanobacteria-dominated crusts have harsh environmental adaptations and are, therefore, dominant in extreme environments (Belnap et al., 2016). Research results from many different global habitats, including deserts, plateaus, mountains, savannas, and even polar regions, revealed that cyanobacterial crusts provide important ecosystem services, such as responding to climate change, dust cycling (Rodriguez-Caballero et al., 2022), hydrological change (Li et al., 2018), and enhancement of global carbon sink capacity (Zou et al., 2022). In soil ecosystems, cyanobacteria are usually the pioneers of community succession, providing carbon and nitrogen to the soil (Belnap, 2002; Housman et al., 2006) and promoting soil nutrient cycling. It also prevents soil erosion and reduces water evaporation (Felde et al., 2018), indirectly enhancing soil stability and improving ecosystem quality (Hagemann et al., 2015; Belnap et al., 2016; Chamizo et al., 2016). Soil properties reflect regional climate, bedrock, geological features, and biodiversity. Research has been conducted on the co-changes in cyanobacteria organisms and soil. Cano-Díaz et al. explored cyanobacterial diversity in gypsum soils in central Spain and revealed that gypsum soils are dominated by filamentous cyanobacteria and have lower abundance and diversity (Cano-Díaz et al., 2018). In the mountainous regions of the northern Urals, where weathering is intense, coarse sandy soils dominate steep slopes and are dominated by spherical cyanobacteria, whereas soils in depressions and gentle areas are fine-textured and dominated by filamentous cyanobacteria (Patova et al., 2018). Temraleeva (2018) explored cyanobacteria species diversity in Russian arid and desert steppe semi-desert soils, meadow soils, and chestnut calcareous soils. Roncero-Ramos et al. (2019) explored the structural diversity of cyanobacterial communities developed from clay, silty, and sandy loam of calcareous sandstones and calcareous mudstones in southeastern Spain and revealed the differences in cyanobacterial composition in soils from different geographical regions. However, more studies based on the diversity of BSCs cyanobacteria in different soil matrices are yet to be conducted.

The composition and dominance of cyanobacterial species corresponds to different soil environments. *Microcoleus*, *Scytonema*, *Phormidium*, *Trichocoleus*, *Leptolyngbya,* and *Tychonema* have been widely reported in the desert, with *Microcoleus vaginatus* being overwhelmingly dominant (Hagemann et al., 2015; Zhang et al., 2015; Etemadi-Khah et al., 2017; Fernandes et al., 2018; Zhang et al., 2021; Sosa-Quintero et al., 2022). In temperate arid steppe and dryland ecosystems, cyanobacterial crusts mainly consist of Nostocales, Oscillatoriales, Synechoccocales, and the dominant species beside *M. vaginatus*, including *Symplocastrum purpurascens*, *Scytonema* sp., *Nostoc commune*, *Phormidium* sp., with the biomass of cyanobacteria increasing with light intensity (Büdel et al., 2009; Williams et al., 2016; Büdel et al., 2018; Temraleeva, 2018; Roncero-Ramos et al., 2019). In the mountains of northeastern Europe, cyanobacteria mainly consist of *Phormidium*, *Leptolyngbya*, and *Nostoc*, with *Leptolyngbya voronichiniana*, *Leptolyngbya foveolarum*, *Trichocoleus hospitus* dominating (Gaysina et al., 2018; Novakovskaya et al., 2022). The main cyanobacteria in Brazil’s tropical savanna include *Microcoleus*, *Nostoc*, *Leptolyngbya*, *Porphyrosiphon*, and *Pycnacronema* (Machado de Lima et al., 2019, 2021). In cold polar environments, Chroococcales, Pseudanabanales, and Oscillatoriales are the main cyanobacterial constituent groups but lack genera such as *Oculatella* and *Hassallia*, which are common in temperate and tropical regions and where humidity is high, and are usually covered with thicker *Nostoc* spp. with enhanced nitrogen fixation capacity (Pushkareva et al., 2015, 2016, 2018). Although there are certain similarities in genus composition, the species are different. However, whether there are differences in ecological functions between different cyanobacterial crusts remains unclear because of the lack of taxonomic information on cyanobacteria from different areas. To more accurately interpret their ecological functions from global patterns and apply their restoration value for degraded ecosystems, more attention should be paid to cyanobacteria taxonomy in karst critical zones.

Karst is the sum of the binary three-dimensional structure of landscapes and phenomena between the surface and the ground formed by erosion, dissolution, collapse, and accumulation of the affection by flowing water to soluble rocks. Karst desertification is an extreme process of ecosystem degradation (Ravbar and Šebela, 2015). South China karst is the most comprehensive, complex, and longest-developed karst landscape in the world and is one of the three largest continuous carbonate rock distribution areas worldwide (Chen et al., 2019). The expansion of rock desertification is exacerbated by the uneven spatial and temporal distribution of seasonal precipitation and overloaded economic activities due to high population loads, resulting in shallow and infertile soils, low vegetation coverage, soil erosion, and high rock exposure rates, leading to the degradation of the ecosystem and eventually presenting a karst desertification landscape such as desertification. In the desertification area in South China karst, there are completely different climatic, zonal, and non-zonal soil-forming conditions from the above desertification and temperate areas, with the soil dominated by limestone, dolomite, and other carbonate rocks and zonal yellow and brown soil (Sheng et al., 2016). In this particular context, studies based on the synergistic evolution of cyanobacteria crust species diversity and soil properties in karst desertification are still lacking. This study examined the diversity and composition of the extant cyanobacteria crust species in the three karst desertification study areas. The synergistic evolutionary relationship with cyanobacteria is discussed in terms of different habitats and soil physicochemical properties, which in turn provides data support for the study of cyanobacterial crust soils in karst desertification areas, a reference for ecological restoration of karst desertification soils, and a case study of karst desertification ecological restoration for global desertification.

## 2. Materials and methods

### 2.1. Study area

**Figure 1:**
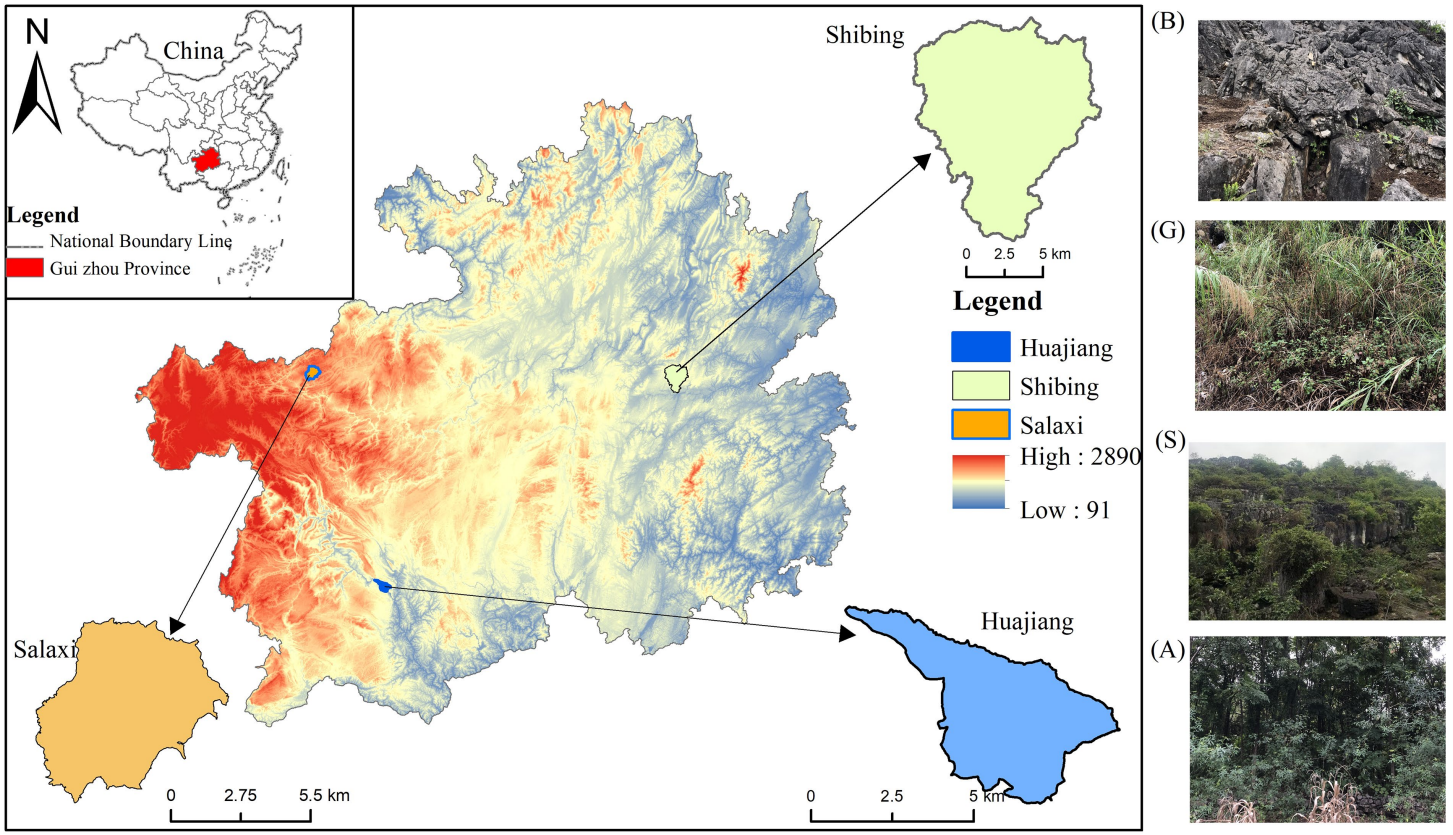
Study area and sampling community. B-Bare land; G-Grassland; S-Shrub land; A-Arbor woodland, same as below.

The Guizhou Plateau was selected as the study area, as it represents the overall ecological structure of South China karst, and chose the Guanling-Zhenfeng Huajiang (HJ) study area with moderate–severe desertification, the Bijie Salaxi (SLX) study area with potential-mild desertification, and the Shibing (SB) study area with no-potential desertification (Figure 1). These areas are dominated by a subtropical humid monsoon climate with rain and heat during the same period but retain differences in lithology, soils, and vegetation. HJ is a typical plateau canyon area with an altitude of 450–1,450 m, an average annual temperature of 18°C, and an average annual precipitation of over 1,200 mm (Zhang et al., 2019). The vegetation type is mainly scrub, broad-leaved forest, and mixed coniferous forest. The altitude of SLX ranges from 1,410 to 1780 m, the average annual temperature is 12°C, the average annual precipitation is above 984.40 mm, and the vegetation type is dominated by scrubs, broad-leaved forests, and coniferous forests. SB is a typical dolomite karst area with an altitude of 526–1,576 m, an average annual temperature of 16°C, an average annual precipitation of 1,110 mm or more, and a vegetation type mainly comprised of subtropical evergreen broad-leaved forests (Wang et al., 2019); the differences in soil properties are shown in Table 1.

**Table 1:** Difference of soil factors in the study area.

	HJ	SLX	SB
Soil parent material	Limestone	Limestone, Sand shale	Dolomite
Main components	Calcite, Dolomite, Magnesite, Other carbonate minerals (CaCO_3_)	Calcite, Dolomite, Magnesite, Other carbonate minerals (CaCO_3_)	Dolomite, Quartz, Feldspar, Calcite, Clay mineral (CaMg(CO_3_)_2_)
Soil-forming material	Clay material rich in Al_2_O_3_ and Fe_2_O_3_	Clay material rich in Al_2_O_3_ and Fe_2_O_3_	Clay material rich in CaO and MgO
Soil type	Limestone soil, Sandy loam	Yellow soil, Yellow brown soil, Limestone soil	Black limestone soil, Yellow soil, Yellow brown soil
Soil thickness	12-50 cm	50-70 cm	60-80 cm

### 2.2. Sample sites

Sampling sites were identified in the three study areas, and the vegetation composition of bare land, grassland, shrubland, and arbor woodland, as well as information on key species, latitude, longitude, elevation, coverage, etc., were investigated. The major species and environmental factors of sampling sites of the same community type should be as similar as possible, with a sampling quadrat of 10 m × 10 m for bare land, grassland, and shrubland and 30 m × 30 m for arbor woodland, specific sampling quadrats information are shown in Table 2.

**Table 2:** Sampling point information.

Study area	Community type	Sampling point	Longitude and latitude	Altitude(m)	Coverage	Main vegetation
HJ	Bare land	HB1	25°39 ′3”N; 105°40′25″E	750	/	/
		HB2	25°39 ′3”N; 105°40′25″E	760	/	
		HB3	25°39 ′3”N; 105°40′25″E	740	/	
	Grassland	HG1	25°39 ′59”N; 105°40′2″E	580	70%	*Rhus chinensis* Mill. *Arthraxon hispidus* (Thunb.) Makino *Bidens pilosa* L.
		HG2	25°39 ′52”N; 105°40′5″E	600	75%	
		HG3	25°39 ′41”N; 105°40′15″E	690	60%	
	Shrubland	HS1	25°29 ′20”N; 105°38′34″E	830	90%	*Alchornea trewioides* (Benth.) Muell. Arg. *Zanthoxylum bungeanum* Maxim. *Broussonetia papyrifera* (L.) Vent.
		HS2	25°39 ′21”N; 105°38′34″E	850	85%	
		HS3	25°40 ′6”N; 105°39′15″E	840	85%	
	Arbor woodland	HA1	25°39 ′11”N; 105°40′21″E	770	65%	*Viburnum foetidum* var. *ceanothoides* Hand.Maz. *Ailanthus altissima* (Mill.) Swingle. *Juglans regia* L.
		HA2	25°39 ′11”N; 105°40′21″E	760	60%	
		HA3	25°39 ′19”N; 105°40′32″E	770	60%	
SLX	Bare land	SB1	27°14 ′30”N; 105°5′56″E	1760	/	/
		SB2	27°15 ′20”N; 105°5′12″E	1910	/	
		SB3	27°15 ′21”N; 105°5′14″E	1890	/	
	Grassland	SG1	27°15 ′24”N; 105°5′13″E	1900	60%	*Trifolium repens* L. *Imperata cylindrica* (L.) Beauv. *Arthraxon hispidus* (Thunb.) Makino
		SG2	27°15′23”N; 105°5′13″E	1950	60%	
		SG3	27°15 ′20”N; 105°5′19″E	1910	60%	
	Shrubland	SS1	27°15 ′9”N; 105°5′31″E	1880	90%	*Rosa roxburghii* Tratt. *Pyracantha fortuneana* (Maxim.) Li *Cotoneaster franchetii* Bois
		SS2	27°15 ′0”N; 105°5′37″E	1890	85%	
		SS3	27°14 ′53”N; 105°5′47″E	1870	90%	
	Arbor woodland	SA1	27°14 ′33”N; 105°4′6″E	1920	70%	*Castanea seguinii Dode* *Betula luminifera* H. Winkl. *Populus alba* L.
		SA2	25°39 ′11”N; 105°40′21″E	1850	65%	
		SA3	27°13 ′19”N; 105°4′35″E	1810	65%	
SB	Bare land	BB1	27°6 ′49”N; 108°7′7″E	870	/	/
		BB2	27°6 ′51”N; 108°7′6″E	846	/	
		BB3	27°6 ′51”N; 108°7′6″E	870	/	
	Grassland	BG1	27°4 ′39”N;108°7′28″E	770	75%	*Miscanthus sinensis* *Arthraxon hispidus* (Thunb.) Makino *Erigeron annuus* (L.) Pers
		BG2	27°4 ′39”N; 108°7′28″E	758	80%	
		BG3	27°4 ′39”N; 108°7′28″E	770	80%	
	Shrubland	BS1	27°7 ′24”N; 108°7′31″E	890	70%	*Pyracantha fortuneana* (Maxim.) Li *Spiraea salicifolia* L. *Coriaria nepalensis* Wall
		BS2	27°6 ′56”N; 108°7′2″E	870	75%	
		BS3	27°6 ′56”N; 108°7′2″E	870	80%	
	Arbor woodland	BA1	27°6 ′58”N; 108°6′18″E	900	95%	*Platycarya longipes* Wu *Quercus phillyraeoides* A. Gray *Cinnamomum cassia* Presl
		BA2	27°6 ′58”N; 108°6′20″E	900	95%	
		BA3	27°6 ′58”N; 108°6′51″E	910	95%	

### 2.3. Crust sample collection and procession

#### 2.3.1. Sample collection

In April 2021, BSCs were collected from different communities in the three study areas, 1 cm thickness of cyanobacteria-dominated BSCs was collected extensively and randomly with small shovels in the sampling quadrat, mixed into one sample, and placed in plastic bags. Three mixed samples were collected from each of the four community types, and 12 mixed samples were collected from one study area, resulting in a total of 36 mixed BSCs samples from the three study areas, which were placed in plastic bags and taken back to the laboratory as soon as possible.

#### 2.3.2. Sample preservation and identification

The field-collected BSCs were placed in specimen bottles containing propanetriol-formaldehyde-water solution (1:1:8) fixative and stored at room temperature in the laboratory of the School of Karst Science, Guizhou Normal University, where cyanobacteria species identification, dominant species recording and micrographs were taken using an OLYMPUS-CX41 trinocular microscope (Yan et al., 2021).

Dominant species: a small piece of BSCs was removed with small forceps to make a temporary water mount. Three temporary slices of each sample were taken, and 10 fields of view were observed for each mount to determine the dominant cyanobacteria species based on the frequency of occurrence (Zhang et al., 2008).

### 2.4. Soil sample collection and procession

#### 2.4.1. Sample collection

In each community quadrate, an S-shaped random sampling method was used to collect the physical and chemical properties of different analysis targets; the ring knife sampling method was used for the physical properties of the soil, the soil with two layers of 0–5 cm and 5–10 cm was selected, and chemical properties selected 0–10 cm mixed soil. The soils were taken back to the laboratory to dry naturally, ground, and sieved through 2 and 0.15 mm soil sieves and stored in plastic bags at room temperature for later determination of indicators.

#### 2.4.2. Methods for the determination of physical and chemical indicators

Soil moisture content was determined by the ring-knife and drying methods; pH was determined using the 1:2.5 soil/water ratio centrifugal sedimentation method and a Leici PHS-25 acidity meter; soil texture was determined by the international classification standard, which classifies soil particle size into clay (<0.002 mm), silt (0.002–0.02 mm), fine sand (0.02 ~ 0.2 mm), and coarse sand (0.2 ~ 2 mm), using the hydrometer method; soil organic carbon was determined by the potassium dichromate oxidation-external heating method; soil total nitrogen was determined by sulfuric acid-catalyst digestion and the Kjeldahl nitrogen determination method; available nitrogen was determined by the alkali-hydrolysis diffusion method; soil total phosphorus was determined by the molybdenum antimony spectrophotometric method; soil available phosphorus was determined by sodium fluoride hydrochloric acid leaching and the molybdenum antimony anti-colorimetric method (Bao, 2000).

### 2.5. Data statistics and analysis

#### 2.5.1. Cyanobacterial diversity index

Using the Shannon-Wiener diversity index:


$$H=-\overset{s}{\underset{i=1}{\sum }}Pi\log_{2}Pi$$

where *S* is the number of species in cyanobacteria,Pi is the proportion of individuals of the species among all individuals, and *H* is the diversity index.

#### 2.5.2. Data analysis

The data were processed and analyzed using Excel 2013 and SPSS 22.0. One-way analysis of variance (ANOVA) and principal component analysis were used to analyze and test the soil physicochemical properties and cyanobacterial diversity using Canoco 5 software for redundancy analysis and plotting, and the remaining graphs were plotted using the Origin 2021 software.

## 3. Results

### 3.1. Taxa and diversity characteristics of cyanobacteria in karst desertification areas

#### 3.1.1. Cyanobacteria taxa

**Figure 2:**
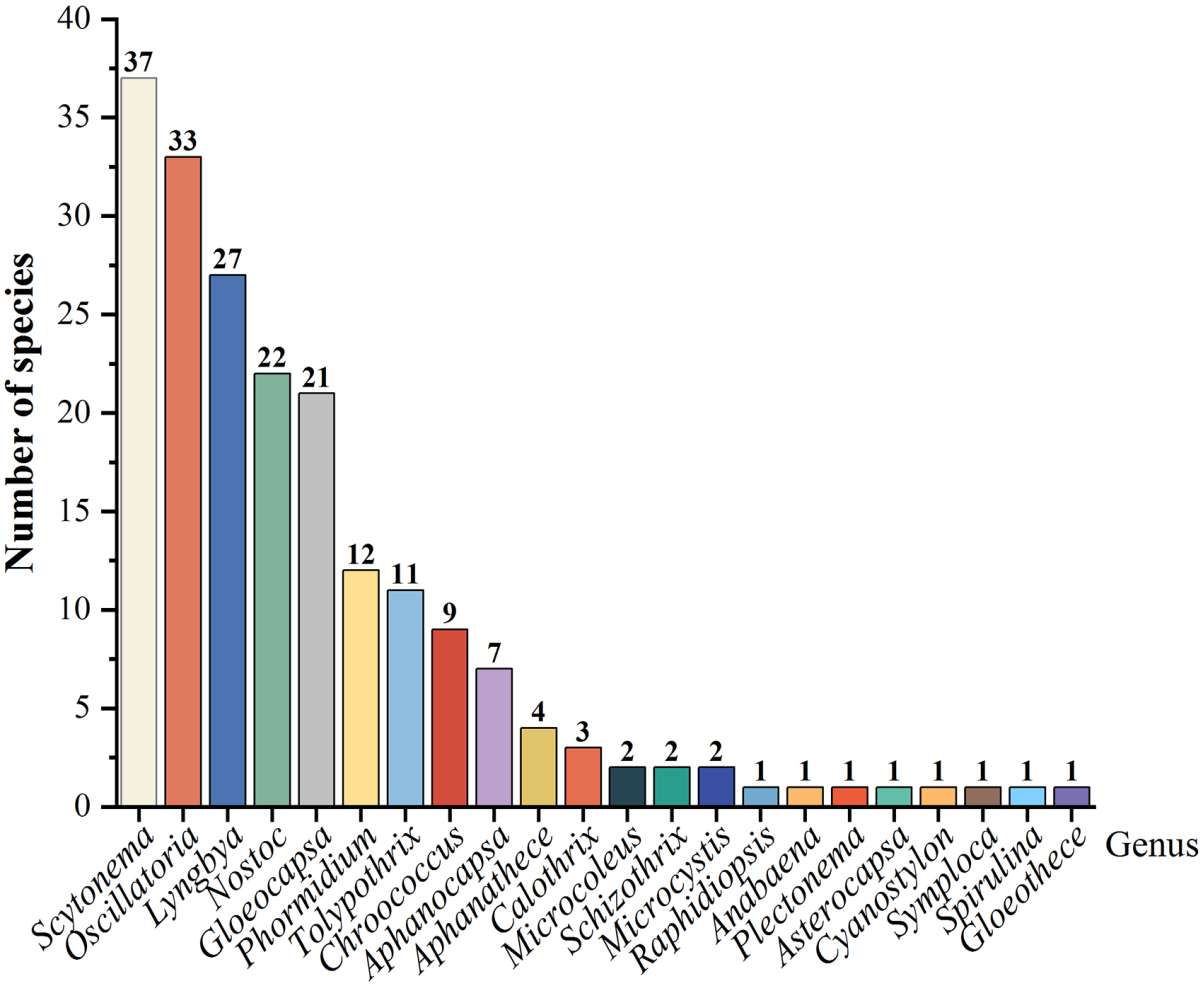
Number of Genus and Species of cyanobacteria.

There are 200 species of cyanobacteria in two classes, five orders, six families, and 22 genera in the three karst desertification study areas, with filamentous cyanobacteria accounting for 77.4% of the total number of cyanobacteria. Oscillatoriales (39%) accounted for the largest proportion, followed by Scytonematales (24.5%), Chroococcales (23%), Nostocales (11.5%), and Rivulariales (2%). Oscillatoriaceae and Chroococcaceae were the dominant families and only three genera of Scytonemataceae, but the number of species was second only to that of Chroococcaceae, accounting for 24.5% (Table 3). The dominant genera were *Scytonema*, *Oscillatoria*, *Lyngbya*, *Nostoc*, and *Gloeocapsa*, with filamentous cyanobacteria being overwhelmingly dominant (Figure 2).

**Table 3:** The statistics of the number and proportion of Family, Genus, Species of cyanobacteria.

Plylum	Order	Family	Genus	Proportion of total Genus	Species	Proportion of total Species
Cyanophyta	Oscillatoriales	Oscillatoriaceae	7	31.8%	78	39%
	Scytonematales	Scytonemataceae	3	13.6%	49	24.5%
	Chroococcales	Chroococcaceae	7	31.8%	45	22.5%
		Cyanostylonaceae	1	4.6%	1	0.5%
	Nostocales	Nostocaceae	2	9.1%	23	11.5%
	Rivulariales	Rivulariaceae	2	9.1%	4	2%
Total	5	6	22	100%	200	100%

#### 3.1.2. Composition and distribution for each study area

**Figure 3:**
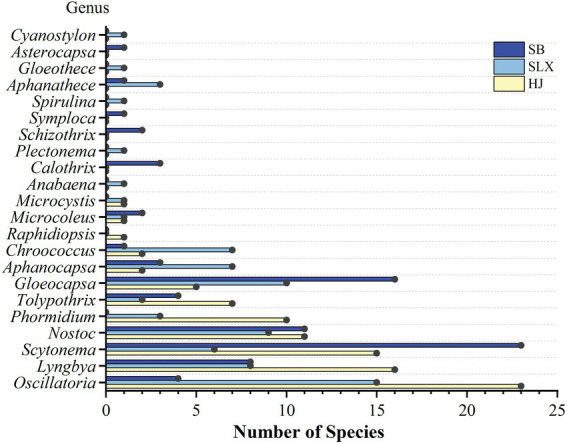
Species number of Cyanobacteria.

The distribution and dominance of cyanobacteria families and genera differed among the different karst desertification study areas (Table 4). A total of 94 species of cyanobacteria from 5 families and 12 genera were found in HJ, with Oscillatoriaceae, the most dominant family, accounting for 54.25% of the total number of species, and Scytonemataceae, the subdominant family, accounting for 23.40%. The dominant genera were *Oscillatoria* (23), *Lyngbya* (16), and *Scytonema* (15). A total of 77 species of cyanobacteria from 5 families and 17 genera were found in SLX, with Chroococcaceae being the most dominant, accounting for 37.66% of the total number of species. Oscillatoriaceae accounted for 36.36% of the total number of species. The dominant genera were *Oscillatoria* (15), *Gloeocapsa* (10), and *Nostoc* (9). There were 80 species of cyanobacteria in 5 families and 14 genera in SB, with Scytonemataceae being the most dominant, accounting for 33.75% of the total number of species, and Chroococcaceae being subdominant, accounting for 27.50%. The dominant genera were *Scytonema* (23), *Gloeocapsa* (16), and *Nostoc* (11) (Figure 3).

**Table 4:** Family and genus compsition of Cyanobacteria

Phylum	Families	Genera	HJ				SLX				SB			
			*B*	*G*	*S*	*A*	*B*	*G*	*S*	*A*	*B*	*G*	*S*	*A*
Cyanophyta	Oscillatoriaceae	*Oscillatoria*	+	+	+	+	+	+	+	+	+			+
		*Lyngbya*	+	+	+	+	+	+	+	+	+		+	+
		*Microcoleus*	+	+	+	+	+	+	+	+	+	+	+	+
		*Phormidium*	+		+	+	+	+						
		*Schizothrix*									+			
		*Symploca*										+		
		*Spirulina*					+							
	Scytonemataceae	*Tolypothrix*	+	+		+			+			+	+	+
		*Scytonema*	+	+	+	+	+	+	+	+	+	+	+	+
		*Plectonema*							+					
	Chroococcaceae	*Gloeocapsa*		+		+	+	+	+		+	+	+	+
		*Aphanocapsa*				+		+	+		+		+	+
		*Aphanathece*						+	+			+		
		*Gloeothece*							+					
		*Chroococcus*			+	+		+	+				+	
		*Asterocapsa*							+					+
		*Microcystis*		+					+	+				
	Cyanostylonaceae	*Cyanostylon*								+				
	Nostocaceae	*Nostoc*	+	+	+	+	+	+	+	+	+	+	+	+
		*Anabaena*							+					
	Rivulariaceae	*Calothrix*									+		+	+
		*Raphidiopsis*				+								
		Total	7	8	7	11	8	10	15	7	9	7	9	10

The presence of genus in communities in study areas is considered to be +, and absence is blank.

#### 3.1.3. Number of cyanobacteria species

**Figure 4:**
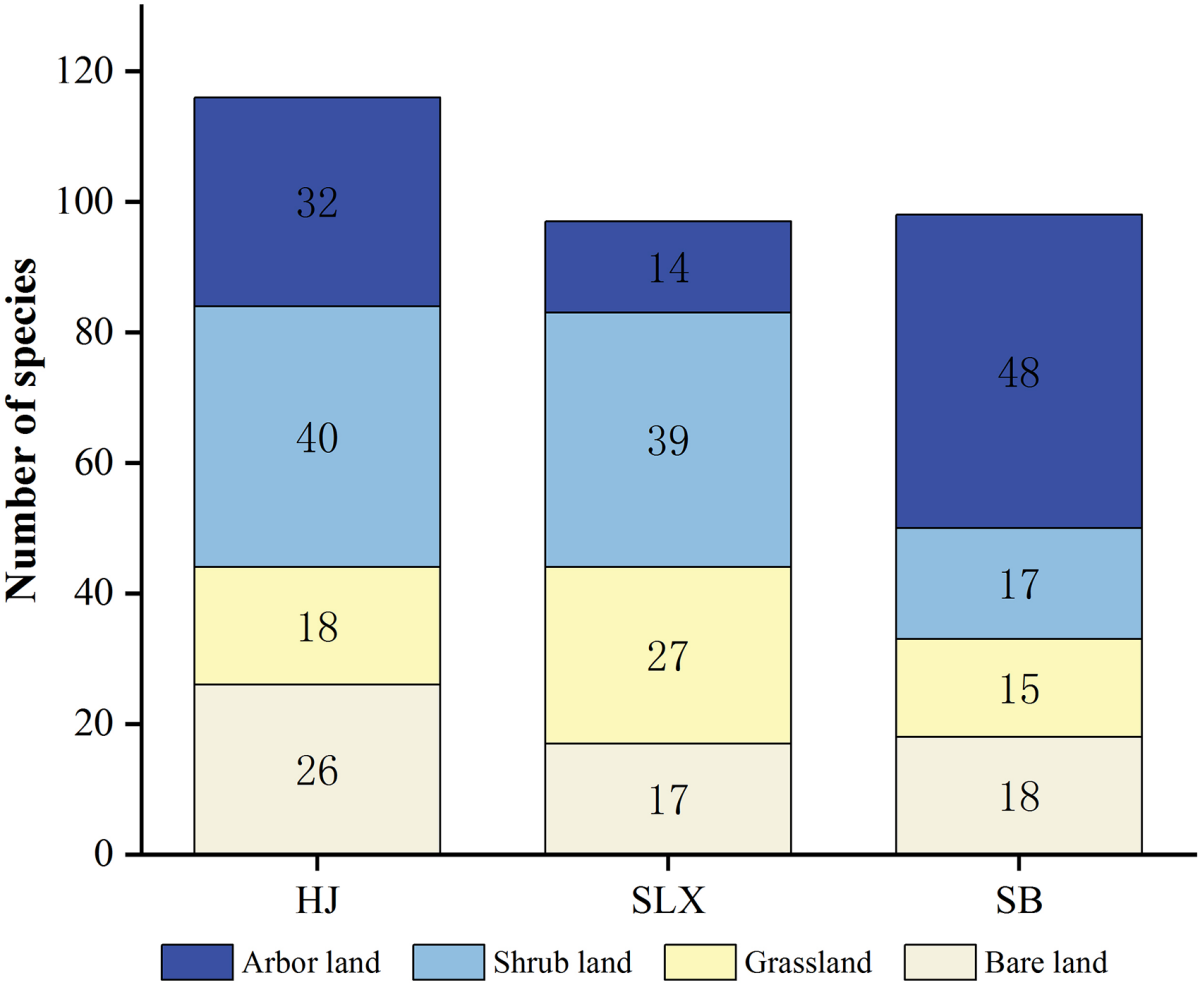
Species number of Cyanobacteria in different community types.

Under the four community habitat types in the three different desertification study areas (HJ, SLX, and SB), there were significant differences in the number of species and genera as the communities evolved from bare land, grassland, shrubland, and arbor woodland (Figure 4). HJ and SLX had the highest number of species in shrubland, with an average cover of over 85% of the community vegetation and 65% of the arbor woodland. The highest number of species was found in the arbor woodland in SB, with an average vegetation cover of 95%, whereas the other communities did not differ significantly. The number of cyanobacteria genera was highest in HJ and SB arbor woodlands and highest in SLX shrubs (Table 4).

#### 3.1.4. Cyanobacterial diversity

**Figure 5:**
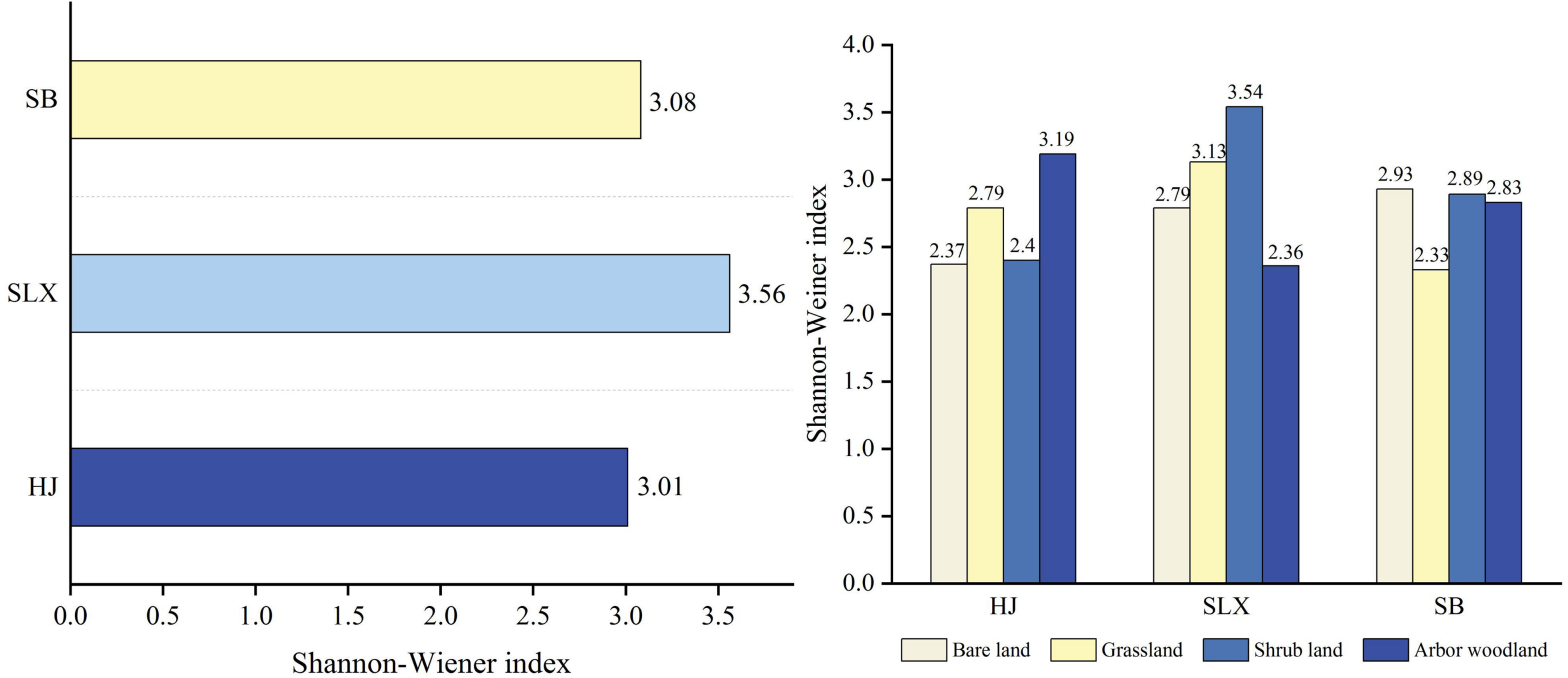
Shannon-Wiener diversity index of cyanobacteria in the study area and different community type.

The Shannon-Wiener diversity index of cyanobacteria differed among the three study areas (Figure 5), with SLX having the highest diversity index, followed by SB and HJ having the lowest, at 3.56, 3.08, and 3.01, respectively. Second, the diversity index also differed among the different types of communities in the three study areas, with HJ having the highest diversity in arbor woodland (3.19), SLX having the highest diversity in shrubland (3.54), and SB having the highest diversity in bare land (2.93).

#### 3.1.5. Dominant species

The dominant species differed among the three study areas and the different community types, with a total of 15 dominant cyanobacteria species identified (Table 5). The dominant species in the three study areas belonged to the genera *Oscillatoria*, *Scytonema*, and *Gloeocapsa*; in HJ, the dominant species were *Scytonema javanicum* and *Lyngbya gracilis*; in SLX, the dominant species were *Lyngbya contorta* and *Gloeocapsa montana,* and in SB, the dominant species were *Scytonema hofmanni* and *Gloeocapsa montana*.

**Table 5:** Dominant species of Cyanobacteria.

Dominant Species	HJ				SLX				SB			
	*B*	*G*	*S*	*A*	*B*	*G*	*S*	*A*	*B*	*G*	*S*	*A*
*Nostoc sp*					+	++	++	++				
*Nostoc commune*	++	++	+	+	++	++	++	++	++	+	++	++
*Scytonema javanicum*			+	+++								
*Scytonema saleyeriense*										++		
*Scytonema hofmanni*			++					+++				+++
*Scytonema julianum*												++
*Scytonema holstii*	++									+		
*Scytonema tenue*				++								
*Scytonema cincinnatum*			++									
*Lyngbya gracilis*			+++									
*Lyngbya contorta*					+++	+++	+	+				
*Microcoleus vaginatus*	+++	+++	++	++	++	++	+++	++	++	+++	++	+++
*Gloeocapsa montana*						+	+++		+++		+++	
*Gloeocapsa alpina*							++					
*Aphanocapsa banaresensis*									++		++	

+++stands for dominant species, ++stands for subdominant species.

The dominant cyanobacteria in all communities were *M. vaginatus* and *Nostoc commune*, *Scytonema* was dominant in the arbor woodlands of all three study areas, *Gloeocapsa* was dominant in the shrublands of SLX and SB, and *Lyngbya* was dominant in the shrubland of HJ, with different dominant species in the grassland and bare land.

### 3.2. Physicochemical properties of soils

#### 3.2.1. Soil moisture content

There were significant differences (*p < 0.05*) in the soil physicochemical indicators between the different desertification study areas, as shown in Table 6. In the limestone-dominated study areas of HJ and SLX, the overall soil moisture content of SLX was greater than that of HJ, and the surface moisture content (0–5 cm) was greater than that of the lower layer (5–10 cm). The difference in soil moisture content between the four different community habitats was not significant, with SLX having the highest moisture content in grassland and HJ having the highest moisture content in shrubland. The dolomite-dominated study area of SB showed that the surface moisture content (0–5 cm) was less than the lower (5–10 cm) surface moisture content, with significant differences in surface moisture content and non-significant differences in lower moisture content in the community habitats, with the highest moisture content in the arbor woodland.

**Table 6:** Soil physicochemical properties.

	HJ				SLX				SB			
**Soil properties**	*B* Mean ± S.E	*G* Mean ± S.E	*S* Mean ± S.E	*A* Mean ± S.E	*B* Mean ± S.E	*G* Mean ± S.E	*S* Mean ± S.E	*A* Mean ± S.E	*B* Mean ± S.E	*G* Mean ± S.E	*S* Mean ± S.E	*A* Mean ± S.E
Wc 0–5 (%)	6.52 ± 0.26a	6.81 ± 0.63a	14.94 ± 4.90a	9.72 ± 1.40a	20.00 ± 1.34a	24.57 ± 4.06a	21.27 ± 2.18a	21.33 ± 1.38a	7.17 ± 0.27b	5.32 ± 0.98b	8.93 ± 0.42ab	15.72 ± 4.56a
Wc 5–10(%)	6.02 ± 0.92a	7.05 ± 0.79a	12.41 ± 3.18a	8.08 ± 1.69a	19.98 ± 1.87a	18.37 ± 5.75a	19.52 ± 1.96a	19.23 ± 1.87a	7.70 ± 0.41a	6.03 ± 1.01a	10.74 ± 2.32a	15.99 ± 5.88a
pH	6.47 ± 0.12a	6.61 ± 0.14a	6.75 ± 0.07a	6.48 ± 0.21a	6.16 ± 0.17a	6.14 ± 0.18a	6.1 ± 0.24a	6.28 ± 0.13a	6.14 ± 0.13a	6.02 ± 0.26a	5.93 ± 0.12a	6.08 ± 0.18a
Cs(%)	5.08 ± 0.65a	4.32 ± 0.80a	4.87 ± 0.73a	5.72 ± 1.81a	28.20 ± 4.12ab	18.16 ± 1.07bc	16.00 ± 1.50c	30.11 ± 4.71a	12.29 ± 1.20a	14.33 ± 3.05a	16.52 ± 3.96a	12.44 ± 5.39a
Fs(%)	63.81 ± 4.20a	64.57 ± 3.39a	72.15 ± 3.0a	65.44 ± 2.0a	33.49 ± 7.65b	54.86 ± 2.61a	52.35 ± 1.33a	41.57 ± 4.23ab	61.4 ± 0.62a	49.35 ± 1.34c	55.83 ± 1.11b	46.58 ± 0.39c
S(%)	23.33 ± 4.06a	24 ± 3.06a	15.33 ± 2.67a	22 ± 1.15a	29.33 ± 3.53a	20 ± 3.06b	23.33 ± 1.33ab	20.67 ± 0.67b	16.67 ± 1.33b	27.33 ± 1.33ab	18 ± 4.16b	31.33 ± 4.81a
Sc(%)	7.78 ± 0.83a	7.11 ± 0.93a	7.65 ± 0.67a	6.85 ± 0.67a	8.98 ± 0a	6.98 ± 0b	8.31 ± 0.67ab	7.65 ± 0.67ab	9.65 ± 0.67a	8.98 ± 0a	9.65 ± 0.67a	9.65 ± 0.67a
SOC(g/kg)	25.60 ± 6.78a	14.61 ± 3.58a	29.56 ± 2.01a	30.60 ± 7.21a	20.74 ± 3.18a	28.98 ± 2.81a	24.73 ± 4.92a	20.63 ± 6.87a	27.84 ± 1.18a	14.34 ± 7.29a	12.53 ± 1.64a	30.94 ± 14.2a
TP(g/kg)	1.5 ± 0.62a	0.80 ± 0.12a	0.52 ± 0.30a	2.49 ± 0.84a	0.98 ± 0.20a	1.51 ± 0.47a	0.84 ± 0.07a	0.8 ± 0.21a	0.58 ± 0.08a	0.30 ± 0.06b	0.37 ± 0.06b	0.17 ± 0.04b
AP(mg/kg)	5.17 ± 3.64ab	1.48 ± 0.61b	1.47 ± 0.11b	10.02 ± 5.93a	5.58 ± 1.31a	1.32 ± 0.10a	7.87 ± 5.38a	1.92 ± 1.06a	1.42 ± 0.80a	2.23 ± 1.54a	0.39 ± 0.02a	1.73 ± 0.54a
TN(g/kg)	3.58 ± 0.59a	2.69 ± 0.35a	3.93 ± 0.27a	3.75 ± 0.60a	2.44 ± 0.32a	3.37 ± 0.58a	2.76 ± 0.53a	1.97 ± 0.58a	2.80 ± 0.15a	1.45 ± 0.67a	1.51 ± 0.19a	1.99 ± 0.68a
AN(g/kg)	0.35 ± 0.07b	0.22 ± 0.04b	0.45 ± 0.02a	0.4 ± 0.08ab	0.32 ± 0.04a	0.48 ± 0.08a	0.37 ± 0.09a	0.28 ± 0.07a	0.36 ± 0.03a	0.20 ± 0.10a	0.19 ± 0.02a	0.30 ± 0.11a

The data in the table represent the mean ± standard deviation; the letters a, b, c indicate the significant difference (*p* < 0.05) between different communities (bare land, grassland, shrubland, and arbor woodland) in the study area. Wc-Soil moisture content; Cs-Coarse sand; Fs-Fine sand; S-Silt; Sc-Soil clay; SOC-Soil organic carbon; TP-Total phosphorus; AP-Available phosphorus; TN-Total nitrogen; AN-Available nitrogen, same as below.

#### 3.2.2. pH and soil texture

The pH of the selected karst desertification study area was neutral to acidic, with overall non-significant differences ranging from 5.93 to 6.75. The soil particles were mainly composed of sand, with the proportion of fine sand content being the largest among the soil particles in all three study areas, with an average content of HJ (66.49%) > SB (53.29%) > SLX (45.57%), with significant differences in the SLX and SB community habitats. The coarse sand content averaged 14% in the three study areas, showing SLX (23.12%) > SB (13.90%) > HJ (4.99%), with no significant differences between the HJ and SB community habitats. There was no significant difference in silt particles in the HJ community habitat, but there were significant differences in the SLX and SB community habitats, with the largest content in the SB arbor woodland and the lowest in the HJ shrubland. The clay content was below 10% in all the study areas.

#### 3.2.3. Organic carbon, phosphorus, and nitrogen

In terms of soil nutrients, the average content of organic carbon, total phosphorus, and total nitrogen in all three study areas showed a maximum in HJ, followed by SLX, and a minimum in SB. The available nitrogen content was generally uniform with little variation. The organic carbon and total nitrogen contents did not differ significantly among the communities in the three study areas, and the average content was more uniform within the community habitats. The differences in total phosphorus content were significant in the SB communities but not in the HJ and SLX communities. The differences in available phosphorus were not significant in the communities of SLX and SB but significant in the communities of HJ, with the highest value in the arbor woodland of HJ and the lowest value in the shrubland of SB, whereas the differences in available nitrogen were not significant in the communities of SLX and SB.

### 3.3. Cyanobacteria species diversity and soil properties

#### 3.3.1. Soil property factors affecting the cyanobacteria species diversity

**Figure 6:**
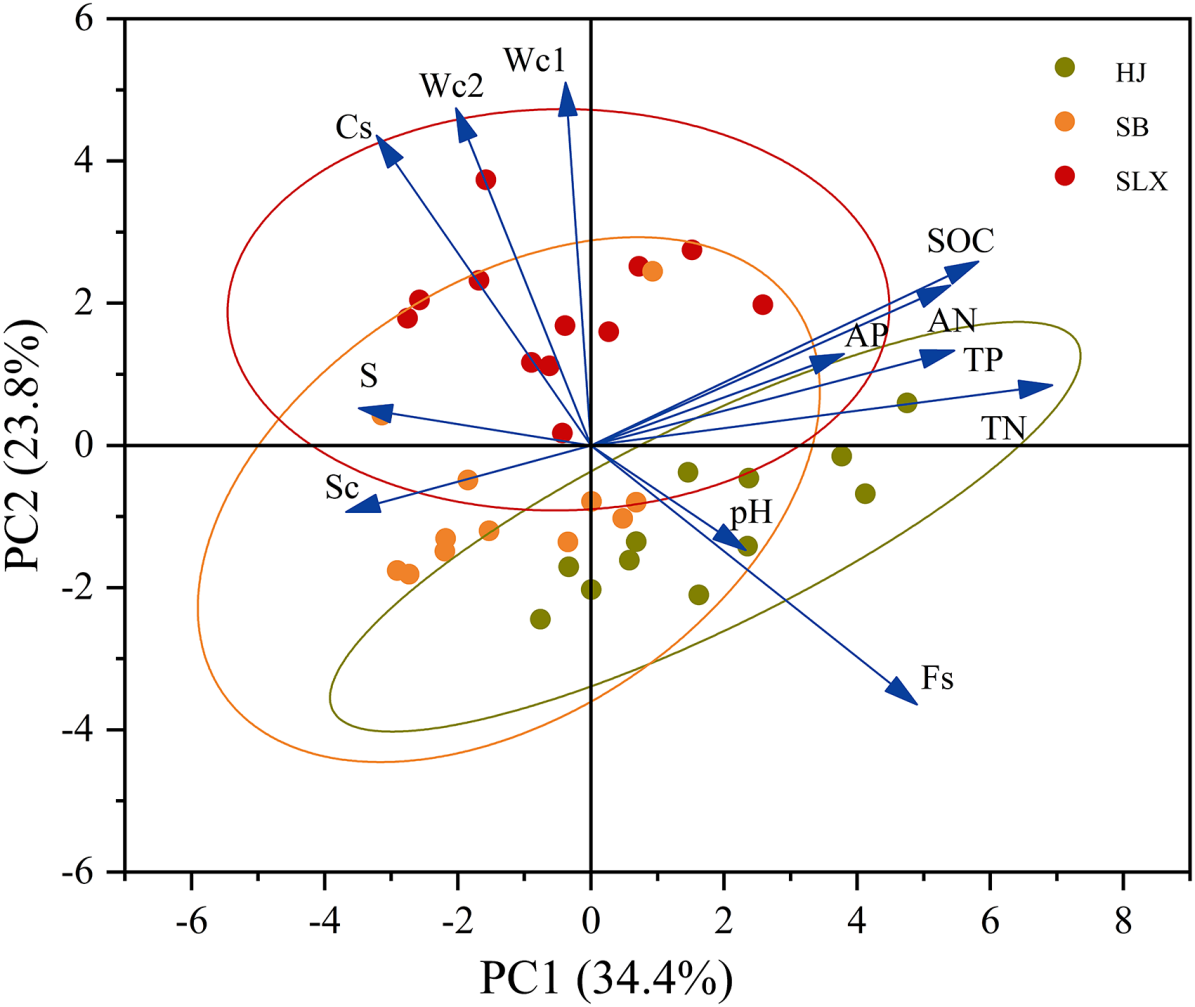
Principal component analysis of soil properties.

Principal component analysis (PCA) was conducted on 12 soil property factors for each community in the three study areas, with the aim of screening out the principal components that may influence cyanobacteria species diversity. The results of the PCA analysis of the soil property factors in the study areas showed that PC1 explained 34.4% of the soil property factors and PC2 explained 23.8% of the soil property factors, with differences between the soil property factors in the three study areas (Figure 6). The first principal component explained the soil property factors affecting cyanobacteria species diversity as organic carbon, total nitrogen, total phosphorus, available nitrogen, and available phosphorus, whereas the second principal component explained soil moisture content (0–5 cm, 5–10 cm) and coarse and silt in the soil particle composition, thus excluding the three factors of fine sand, clay, and pH.

#### 3.3.2. Redundancy analysis of cyanobacterial diversity and soil properties

**Figure 7:**
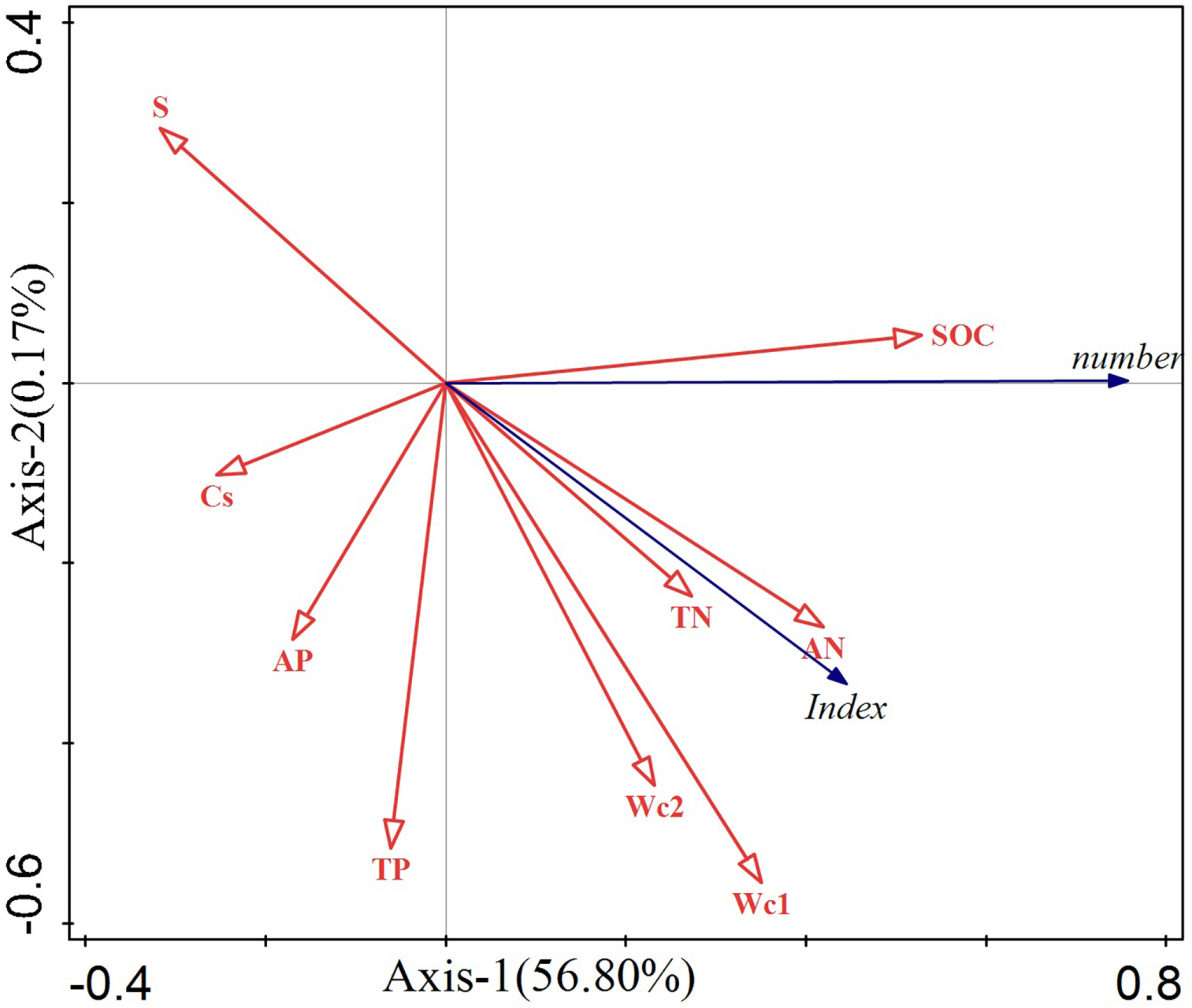
Redundancy analysis of cyanobacteria diversity and soil properties.

To clarify the relationship between cyanobacterial diversity and soil properties, a redundancy analysis was conducted by combining the cyanobacterial diversity index and the number of species with nine soil property factors (Figure 7). The results showed that Axis-1 explained 56.8% of the results, with organic carbon, total nitrogen, available nitrogen, soil moisture content (0–5 cm), and soil moisture content (5–10 cm) all pointing to Axis-1. There were positive correlations (number and index) between the response factors, with the number of cyanobacteria species mainly influenced by organic carbon (contribution of 27.9%), with a significant positive correlation with organic carbon (*p* < =0.03). The diversity index was mainly influenced by soil moisture content (0–5 cm) (contribution of 23%), with a significant positive correlation with soil water content (0–5 cm) (*p* < =0.012), and the diversity index was also influenced by total nitrogen (contribution of 19.6%), with a significant positive correlation with total nitrogen (*p* < =0.016). Although soil moisture content (5–10 cm) and moisture nitrogen also pointed to the same axis, indicating that there may also be an effect on cyanobacteria species diversity, they did not show statistical significance in the analyzes performed (*p* > 0.05).

## 4. Discussion

### 4.1. Similarities and differences in cyanobacterial diversity between karst desertification areas and desertification areas

The BSCs cyanobacteria in the karst desertification study areas were dominated by filamentous cyanobacteria, which accounted for 77.4% of the total. The main families and genera of cyanobacteria are Oscillatoriaceae (*Oscillatoria*, *Lyngbya*), Scytonemataceae (*Scytonema*), Nostocaceae (*Nostoc*), and Chroococcaceae (*Gloeocapsa*), the most common of which is *M. vaginatus* (Garcia-Pichel et al., 2001; Belnap and Lange, 2003; Zhang et al., 2015). In our study, *M. vaginatus* occurred at all sampling sites, which was generally consistent with the composition of cyanobacteria in most other types of desertification areas (Zhang et al., 2005a,b; Büdel et al., 2009; Etemadi-Khah et al., 2017; Cano-Díaz et al., 2018; Roncero-Ramos et al., 2019), indicating that *M. vaginatus* is the main cyanobacteria that make up the BSCs and plays an important ecological role, while *N. commune* also occurred in almost all sample sites and has been shown to occur most frequently among cyanobacteria (Starks et al., 1981). The cyanobacteria in this study are common to the desert ecological zone and have a wide ecological range, which again supports the fact that they are an important genus of cyanobacteria that form BSCs in the desert ecological zone, and also reflects the similarity in the cyanobacterial composition of BSCs in the karst desertification areas as a degraded desert ecological zone. There are also differences, with the genera unique to karst desertification areas being *Cyanostylon* and *Symploca*, with also differences in species, which may be related to differences in regional microhabitats and the adaptation of different cyanobacteria. Desert ecosystems are often considered to be harsh and lifeless habitats, and cyanobacteria can adapt to such extreme environmental conditions and occupy a unique ecological niche (Whitton and Potts, 2007; Perera et al., 2018), as opposed to harsh desertification areas with strong solar radiation, sandy winds, and lack of water. The warm and humid conditions in karst desertification areas provide good conditions for the growth and reproduction of filamentous cyanobacteria, and filamentous cyanobacteria are dominant in cementing fixed soil particles, which can slow down the soil loss caused by the special geomorphic structure in karst areas, improve soil stability, and have important functions in karst soil ecological restoration.

Although the species and numbers of cyanobacteria identified in the three karst desertification study areas are more convincing in this study, the classification of cyanobacteria is based only on morphological microscopic identification. It has been pointed out that the taxonomy of cyanobacteria traditionally relies heavily on morphological data, but the evolutionary relationships of cyanobacteria are more complex, and the true diversity of cyanobacteria is likely to be underestimated (Dvořák et al., 2017). The existing methods also include microscopic identification combined with culture identification, but there are cases where some cyanobacteria are not viable in culture (Dvořák et al., 2018). With the expansion of cyanobacterial gene pools and rapid development of molecular techniques, studies have shown that comprehensive evaluation of cyanobacteria in BSCs requires a combination of multiple molecular techniques, such as DNA extraction, PCR amplification, and sequencing. Molecular tools are often heavily applied to desert BSCs cyanobacteria, and some scholars have used phylogenies to study the global distribution of desert cyanobacteria, concluding that the global distribution of desert cyanobacteria is not the result of widespread dispersal but rather an ancient genetic evolution (Bahl et al., 2011). Other scholars have also argued that cyanobacterial diversity and distribution are determined by complex interactions with multiple abiotic stressors (Lacap-Bugler et al., 2017). However, it is also important to combine a multiphase approach to species description with morphology, as gene sequences can also appear to be improperly assigned to genera (Muhlsteinova et al., 2014), even though the multiphase approach does not guarantee that all cyanobacteria will be identified (Redfield et al., 2002). Therefore, in future research on karst cyanobacteria, the three karst desertification regions also need to combine a multiphase approach to further illustrate the cyanobacterial diversity of the three sites and their intrinsic connections at the geographic scale and phylogeny.

### 4.2. Differences in cyanobacteria species diversity in different karst desertification areas and community habitats

In the three karst desertification study areas, there were differences in the abundance, diversity, and species composition of cyanobacteria. The overall number of species was HJ > SB > SLX, and the diversity index was SLX > SB > HJ. In the same limestone lithological background, the number of species was HJ > SLX, and the diversity index was SLX > HJ. Scholars have shown that cyanobacterial community structure is associated with different physicochemical properties of the colonized mineral matrix and petrographic properties (Khomutovska et al., 2020). The number of cyanobacteria was the highest, and the diversity index was the lowest in the HJ moderate–severe desertification study area, with the largest proportion of Oscillatoriaceae, and the species composition was relatively simple, which is consistent with the conclusion of Yang et al. (2013), and may be related to the hydrothermal combination conditions caused by the microclimate in the region since the study area of HJ in moderate–severe desertification is located in a dry and hot valley with high annual rainfall and evaporation, and high temperature, hence it is dominated by Oscillatoriaceae which is non-heterocystic, swimmable and can grow and develop while avoiding the strong sunlight and dry environment (Belnap et al., 1993). The largest number of cyanobacteria species and the highest diversity index in the SLX potential-mild desertification study area were dominated by Chroococcaceae because of the predominance of spherical cyanobacteria species with greater resilience in SLX at an average altitude of more than 1,000 m, with low mean annual temperatures and a wet and cold environment (Pushkareva et al., 2015). In the background of dolomite lithology, the number of species in the SB study area was second to that of HJ, and the diversity index was lower than that of SLX and dominated by Scytonemataceae, which is consistent with the conclusion of Yan et al. (2021) that the terrestrial algae of the dolomite karst of SB are dominated by Scytonemataceae, reflecting the richness in calcium and magnesium and poverty in nutrients of the dolomite karst and supporting the view that dolomite dominates the growth and development of heteromorphic filamentous cyanobacteria (Hu et al., 2002).

In the four community habitats of three different karst desertification study areas, in terms of species numbers, the number of species in the HJ study area was shrubland > arbor woodland > bare land > grassland; in the SLX study area, shrubland > grassland > bare land > arbor woodland, and the trends were basically the same in the two desertification study areas of the same lithology, except for the opposite positions in the number of arbor woodland and grassland. The number of species in the arbor woodland was the highest in the SB study area, whereas the rest of the communities showed little difference. In terms of diversity indices, HJ had the highest diversity in grassland and woodland, SLX had the highest diversity in grassland and shrubland, and SB had the highest diversity in bare land and shrubland. In summary, it is clear that the number of species in the study area is highest in shrubland and highest in grassland in the lithologically identical HJ and SLX study areas. The highest number of species in the dolomite study area of SB is in the arbor woodland, and the highest diversity is in the bare land, which is not only influenced by lithology, general environment, and soils but also by other factors. It has been suggested that grassland has a greater capacity to trap surface water and that vegetation coverage also affects light intensity and, thus, cyanobacterial diversity (Vijayan and Ray, 2015), combined with the community coverage information in this study.

### 4.3. The influence of karst desertification soil properties on cyanobacterial diversity and species composition

Soil physicochemical properties are influenced by topographic and climatic conditions, bedrock properties, land use, community vegetation, and habitat degradation, resulting in the spatial heterogeneity of regional soil properties (Quesada et al., 1998; Řeháková et al., 2011; Hakkoum et al., 2020). Karst desertification succession is considered to be an extreme land degradation process; however, in our study, soil nutrient content did not decrease with increasing desertification, in agreement with the conclusions reached by Sheng et al. (2016). The organic carbon, total nitrogen, and total phosphorus contents were greatest in the study area of moderate–severe desertification in HJ, followed by SLX, and lowest in SB, due to the increase in the rate of rock exposure as the degree of rock desertification deepened; the nutrients from atmospheric deposition, rock dissolution, and plant apoptosis cannot be absorbed and preserved by the exposed rocks and can only be washed by rain or blown by the wind into thxe surrounding soil, so the soil nutrient content increases, the soil that can be lost is limited too, and nutrient losses are minimal. The results of our study show that the soil properties that affect the cyanobacteria species diversity in the three karst desertification study areas are soil moisture content (0–5 cm), organic carbon, and total nitrogen. Organic carbon is not only positive for the growth and development of cyanobacterial crusts, but also a key factor in promoting the formation of cyanobacterial communities. Some scholars have studied the structure of Arctic cyanobacterial communities and their bearing soil substrates, showing a significant positive correlation between organic carbon and cyanobacteria abundance (Pushkareva et al., 2016). In a study of the community structure of BSCs cyanobacteria in the Tengger East Desert, Zhang et al. (2021) concluded that total nitrogen and organic carbon were the factors that significantly influenced cyanobacterial diversity and abundance, and that the highest organic carbon content and the highest number of cyanobacteria species were found in the arbor woodland of SB, while the highest diversity and total nitrogen content were found in the bare land, thus supporting this conclusion. In studying the relationship between cyanobacterial abundance and rainfall in the Atacama Desert, researchers have concluded that cyanobacterial abundance decreases with decreasing rainfall (Warren-Rhodes et al., 2006). Zhang et al. (2009) concluded that precipitation is the main factor influencing the distribution of cyanobacteria and that the higher the precipitation, the higher the soil moisture content, resulting in a finer soil texture that can support the growth and development of more cyanobacterial communities. In a study of cyanobacteria in BSCs in the Ural Mountains, researchers pointed out that soil moisture content and total nitrogen were important soil property factors affecting cyanobacteria species diversity (Patova et al., 2018), which is generally consistent with the findings of this study. Others have shown that cyanobacterial diversity is most influenced by soil moisture content and less related to soil chemical composition (Hagemann et al., 2015), which is generally consistent with the results of this study. SLX had a significantly higher soil moisture content in all communities than the other two study areas, which, based on the analytical discussion, could be used as an explanation for the highest diversity index in SLX.

### 4.4. Synergistic evolution of cyanobacteria species composition and soil properties in karst desertification areas

The specific physiological and ecological characteristics of cyanobacteria allow them to occupy different ecological niches, and the dominance of cyanobacteria varies depending on habitat and soil conditions (Loza et al., 2014). A study of the cyanobacteria species composition of BSCs in the Saharan desert indicated that *Microcoleus* spp. were significantly dominant in low salinity soils, filamentous heterocystic cyanobacteria and *Acaryochloris* were dominant in high salinity soils, and non-heterocystic cyanobacteria were dominant in dry soils (Mehda et al., 2021), which is consistent with the results obtained by HJ in this study. Cyanobacteria are alkaline lovers that grow best in neutral to slightly alkaline soils (Belnap and Lange, 2003). The soil of the selected communities in karst desertification areas is neutral to acidic, but cyanobacteria species are still abundant, indicating that cyanobacteria can grow and reproduce well in acidic environments. From the analysis of our paper, it can be seen that the soils of HJ are characterized by low soil moisture content (about 7–15%), and sandy loam; the average content of organic carbon is 25.09 and total nitrogen is 3.49; the content of clay is 7.35%, resulting in low soil moisture content supporting the growth of cyanobacteria; SLX soils are characterized by rich soil moisture content (over 17%); the coarse sand content is larger than the other two study areas, and the content of clay is 7.98%, rich in organic carbon and nitrogen, and the soils of SB are characterized by low soil moisture content, similar to HJ, and relatively poor in soil nutrients. The highest organic carbon and total nitrogen content of HJ were dominated by *Oscillatoria* and *Lyngbya*, such as the unique *Oscillatoria animalis* and *Oscillatoria limosa*, etc. In addition, a unique genus *Raphidiopsis* exists. Similarly, the organic carbon and total nitrogen content of SLX was second only to that of HJ, and *Oscillatoria* was also dominant, with the unique species *Oscillatoria prolifica* and *Oscillatoria agardhii*, etc. It can be seen that soils rich in organic carbon and total nitrogen are dominated by cyanobacterial crusts of filamentous non-heterocystic *Oscillatoria*. In contrast, soils with high moisture content and coarse texture were dominated by cyanobacterial crusts of *Gloeocapsa*, such as *Gloeocapsa montana* Kütz and *Gloeocapsa montana* in SLX, in agreement with the conclusions reached by Patova et al. (2018), and the unique genera *Plectonema*, *Anabaena*, *Cyanostylon*, etc. The temperature of SB is suitable, the soil texture is fine and relatively poor in soil moisture and nutrients, and is dominated by cyanobacterial crusts of filamentous heteromorphic *Scytonema*, such as *Scytonema hofmanni* and *Scytonema julianum*, etc. In addition, there also exists the unique *Symploca* and *Calothrix* species.

## 5. Conclusion

1. A total of 200 species of cyanobacteria in two classes, five orders, six families, and 22 genera were identified in the three karst desertification study areas: the species composition of cyanobacteria is similar to that of desertification areas, all of which are common species, with Oscillatoriaceae, Chroococcaceae, Scytonemataceae, and Nostocaceae as common families, among which *M. vaginatus* and *N. commune* are generally dominant, which is related to the wide ecological range of generalist cyanobacteria; the dominant family and genus in the three desertification study areas were different, with Oscillatoriaceae being the dominant family in the moderate–severe desertification study area of HJ, Chroococcaceae in the potential-mild desertification study area of SLX, and Scytonemataceae in the no-potential desertification study area of SB.
2. The diversity of cyanobacteria in the karst desertification study area is related to three soil properties: soil moisture content (0–5 cm), organic carbon, and total nitrogen, but other soil properties affecting the cyanobacterial diversity need to be explored in combination with more soil properties in karst desertification areas. Cyanobacterial diversity varies between community habitats, with the highest number of species in both the HJ and SLX study areas of limestone lithology in shrubland and the highest diversity in grassland. The number of species was highest in arbor woodland, and the diversity was highest in bare land in the dolomite study area of SB. In addition to lithology and soil properties, this is also related to the increase in soil nutrients due to vegetation litter, trapping of surface water, and light intensity directly influenced by vegetation cover.
3. There is a synergistic evolution of suitable cyanobacteria in the karst desertification study area in response to changes in the soil properties. Cyanobacterial crusts of dry and hot soils (7–15% moisture content), and soils rich in organic carbon and total nitrogen, are dominated by *Oscillatoria*, reflecting the physiological adaptability of *Oscillatoria* to dry and hot environments and its carbon and nitrogen-loving properties. Cyanobacterial crusts of *Gloeocapsa* are dominant in soils with high moisture content (above 17%) and coarse texture, reflecting the resistance of *Gloeocapsa* to cold and wet conditions and influenced by coarse soil particles. *Scytonema* is influenced by dolomite lithology and is tolerant of infertile soils at suitable temperatures, so poor nutrients and low moisture content are dominated by *Scytonema* as the dominant cyanobacterial crust.

## Data availability statement

The original contributions presented in the study are included in the article/supplementary material, further inquiries can be directed to the corresponding author.

## Author contributions

QC and NY conceived the work, conducted data sorting, and analysis. NY, QC, and JZ collected the biocrust samples and soil samples. QC performed the microscopic study for cyanobacteria phenotypic characterization. NY performed the soil physicochemical analysis. JZ collected literature. QC wrote the manuscript and NY revised it. KX provided financial support and summarized manuscripts. All authors contributed to the article and approved the submitted version.

## Funding

This research was financially supported by the Key Project of Science and Technology Program of Guizhou Province (No. 5411 2017 Qiankehe Pingtai Rencai); the China Overseas Expertise Introduction Program for Discipline Innovation (No. D17016); the National Major Research and Development Program of China (2016YFC0502607).

## Conflict of interest

The authors declare that the research was conducted in the absence of any commercial or financial relationships that could be construed as a potential conflict of interest.

## Publisher’s note

All claims expressed in this article are solely those of the authors and do not necessarily represent those of their affiliated organizations, or those of the publisher, the editors and the reviewers. Any product that may be evaluated in this article, or claim that may be made by its manufacturer, is not guaranteed or endorsed by the publisher.
